# Energy Storage Performance of Electrode Materials Derived from Manganese Metal–Organic Frameworks

**DOI:** 10.3390/nano14060503

**Published:** 2024-03-11

**Authors:** Gyeongbeom Ryoo, Seon Kyung Kim, Do Kyung Lee, Young-Jin Kim, Yoon Soo Han, Kyung-Hye Jung

**Affiliations:** Department of Advanced Materials and Chemical Engineering, Daegu Catholic University, Gyeonsan-si 38430, Gyeonbuk, Republic of Korea; rkb9770@naver.com (G.R.); seon3193@naver.com (S.K.K.); dokyung@cu.ac.kr (D.K.L.); yjkim@cu.ac.kr (Y.-J.K.); yshancu@cu.ac.cr (Y.S.H.)

**Keywords:** supercapacitors, pseudocapacitors, metal–organic frameworks, manganese oxide, thermal treatment

## Abstract

Metal–organic frameworks (MOFs) are porous materials assembled using metal and organic linkers, showing a high specific surface area and a tunable pore size. Large portions of metal open sites in MOFs can be exposed to electrolyte ions, meaning they have high potential to be used as electrode materials in energy storage devices such as supercapacitors. Also, they can be easily converted into porous metal oxides by heat treatment. In this study, we obtained high energy storage performance by preparing electrode materials through applying heat treatment to manganese MOFs (Mn-MOFs) under air. The chemical and structural properties of synthesized and thermally treated Mn-MOFs were measured by Fourier-transform infrared spectroscopy (FTIR), Raman spectroscopy, X-ray diffraction (XRD), X-ray photoelectron spectroscopy (XPS), and transmission electron microscopy (TEM). The surface area and porosity were investigated by nitrogen adsorption/desorption isotherms. The electrochemical properties were studied by cyclic voltammetry (CV) and galvanostatic charge–discharge (GCD) using a three-electrode cell. It was found that Mn-MOF electrodes that underwent heat treatment at 400 °C under air consisted of Mn_2_O_3_ with high specific surface area and porosity. They also showed a superior specific capacitance of 214.0 F g^−1^ and an energy density value of 29.7 Wh kg^−1^ (at 0.1 A g^−1^) compared to non-treated Mn-MOFs.

## 1. Introduction

Supercapacitors are energy storage devices; their energy storage mechanism is based on the charge distribution at the interface between electrodes and electrolytes, and therefore, they have high power density, fast charging ability, and excellent cycling stability [1,2,3,4]. They consist of three components: electrodes, electrolytes, and separators. Energy storage performance is strongly affected by electrode materials since an energy storage mechanism is determined by these materials [5,6]. Supercapacitors are classified into three categories: electric double-layer capacitors (EDLCs), pseudocapacitors, and hybrid supercapacitors. EDLCs store the charge electrostatically, while pseudocapacitors store the charge electrostatically and electrochemically. Carbon-based nanostructures are widely used as electrode materials in EDLCs because of their high surface area and porosity, promoting the interfacial contact between electrolytes and electrodes. However, these do not deliver satisfactory specific capacitances and energy densities without faradaic redox reactions. Transition metal-based materials (oxides or chalcogenides) are commonly used for pseudocapacitor electrodes, showing high specific capacitances and energy densities caused by reversible redox reactions [7]. A hybrid supercapacitor is an amalgamation of both types.

Applying transition metal oxides to supercapacitor electrodes can enable faradaic redox reactions, resulting in higher energy storage performance. However, their low electrical conductivity and insufficient porosity prevent electrolyte ions from accessing the electrode surface [8]. Among transition metal oxides, manganese oxides are good candidates due to their abundance and environmentally friendly nature, low cost, and excellent capacitance [9,10,11]. Manganese exists as various forms of oxides, such as MnO, MnO_2_, Mn_2_O_3_, or Mn_3_O_4_ with various types of crystal structures. This quality enables a variety of electrochemical properties to be present when manganese oxides are used as electrode materials for energy storage devices, such as lithium ion batteries and supercapacitors [12,13]. Pseudocapacitive performance can be induced by the redox reaction between Mn(III) and Mn(IV). To improve the energy storage performance of manganese oxide electrodes, enlarging the surface porosity is a good strategy, which can be carried out by shortening the transport pathway of electrolyte ions. It was reported that porous manganese oxides can be synthesized via the template method, microemulsion method, hydrothermal method, sonochemical method, or ultrasound irradiation [14].

Metal–organic frameworks (MOFs) are porous materials consisting of metal ions linked together by organic bridging ligands, resulting in large pore volumes and tunable porosities [15,16,17]. These features make them applicable in many areas, such as in catalysts, sensors, and drug delivery [18,19,20,21]. It is also advantageous that transition metal ions in MOFs can serve as redox active sites for electrochemical reactions [22,23,24]. It is well known that the surface area and porosity of electrode materials are crucial for energy storage performance to facilitate electrolyte ions’ access to electrodes for supercapacitors. Applying MOF-based materials to supercapacitor electrodes can be considered a good strategy since their porous structures can promote the access of ions, and transition metal ions enable faradic redox reactions [25,26]. However, it was also reported that insufficient electrical conductivity and poor stability restrict their energy storage performance, and hence, MOF-derived metal oxides have been studied to overcome these defects [27]. It is known that the pore structures of MOFs can be well preserved during thermal treatments, such as calcination, thermal annealing, or thermal decomposition [28]. Additionally, thermal treatment is often employed to obtain well-defined crystalline structures of metal oxides with high purity [29,30,31,32,33]. Applying MOF-derived metal oxides to supercapacitor electrodes is highly promising due to their large surface porosity and improved electrical conductivity [34,35].

In the present study, manganese MOFs were synthesized using terephthalate linkers and thermally treated to obtain high energy storage performance. Their crystalline structures were investigated using X-ray diffraction (XRD) spectroscopy and high-resolution transmission electron microscopy (HR-TEM), while their chemical structures were observed using Fourier-transform infrared (FTIR), Raman, and X-ray photoelectron spectroscopy (XPS). The specific surface area and porosity were obtained by nitrogen adsorption/desorption isotherms. The electrochemical properties were measured by using a three-electrode set using pristine and thermally treated MOFs as working electrodes with 1 M sodium sulfate as an electrolyte.

## 2. Materials and Methods

For the synthesis of manganese MOFs, manganese(II) chloride tetrahydrate (MnCl_2_·4H_2_O), terephthalic acid, and N,N-dimethylformamide (DMAc) were purchased from Tokyo-Chemical Industry (TCI, Tokyo, Japan). To fabricate electrodes, poly(vinyl fluoride) (PVdF, Sigma-Aldrich, St. Louis, MO, USA), Super P carbon black (Alfa Aesar, Haverhill, MA, USA), and 1-methyl-2-pyrrolidinone (NMP, TCI, Tokyo, Japan) were used as a binder, conductive agent, and solvent, respectively. For electrochemical measurements, three-electrode set and sodium sulfate (>99.5%, Na_2_SO_4_) were purchased from Wizmac (Daejeon, Republic of Korea) and TCI, respectively. All chemicals were used as received.

Amounts of 1.2 g MnCl_2_·4H_2_O and 0.8 g terephthalic acid were dissolved in 40 mL DMF, and then 10 mL of methanol was added. This solution was transferred to a Teflon-lined stainless steel autoclave and heated at 120 °C for 24 h. Synthesized MOFs were obtained by centrifugation at 15,000 rpm for 15 min, washing with methanol for three times, and drying in a vacuum at 60 °C for 12 h. These are referred to as Mn-MOFs. To optimize their porosity and electrochemical performance, Mn-MOFs were thermally treated at 400 °C for 30 min under air, and these are represented as A-Mn-MOFs.

A chemical analysis of synthesized and thermally treated Mn-MOFs was conducted using Fourier-transform infrared (FTIR) spectroscopy (ALPHA, Bruker Optics, Billerica, MA, USA). Raman spectroscopy was employed to further study their structures using an inVia reflex, Renishaw, Stonehouse, UK. The crystal structure of MOF-derived manganese oxides was determined by X-ray diffraction (XRD, Phillips PW 3830, Eindhoven, The Netherlands) using Cu-α radiation. Their structures were also characterized by a high-resolution transmission electron microscope (HRTEM, Titan G2 ChemiSTEM Cs Probe, FEI Company, Hillsboro, OR, USA) and energy dispersive spectrum (EDS) for surface morphology and elemental analysis, respectively. X-ray photoelectron spectroscopy (XPS) experiment was performed by using a Thermo Scientific K-Alpha XPS system (Thermo Fisher Scientific, Hemel Hempstead, UK) equipped with a monochromatic Al Ka X-ray source. Surface properties were obtained using nitrogen adsorption/desorption measurement on an BELSORP-max (BEL Japan Inc., Osaka, Japan). Specific surface area was calculated from nitrogen adsorption isotherm using the Brunaure–Emmett–Teller (BET) equation, and pore size distribution was calculated using density functional theory (DFT) methods.

For the measurement of supercapacitor performance, a three-electrode set was assembled using Pt as a counter electrode, Ag/AgCl as a reference electrode, and 1 M Na_2_SO_4_ as an electrolyte. For a working electrode, a blend of Mn-MOFs (pristine or thermally treated), PVdF, and carbon black (7:2:1) dispersed in NMP was placed on the graphite foil by drop-casting and dried in a vacuum at 60 °C for 12 h. Cyclic voltammograms (CVs) and galvanostatic charge/discharge (GCD) testing were conducted on an electrochemical workstation (WBCS3000S, Wonatech, Seoul, Republic of Korea). The specific capacitance (*C*_sp_, F g^−1^) was measured as follows:

(1) $$C_{\mathrm{sp}}\;=\;\frac{I_{d}t_{d}}{mV}$$

where *I*_d_ is the discharge current (A), *t*_d_ is the discharge time (s), *m* is the mass of active material in a working electrode (g), and *V* is the voltage window (V). Cyclic stability was studied by measuring capacitance retention via GCD testing after 5000 cycles at a current density of 1 A g^−1^. Energy density (*E*, Wh kg^−1^) and power density (*P*, W kg^−1^) were calculated using Equations (2) and (3), respectively, as follows:

(2) $$E=\frac{I_{d}t_{d}V}{2m}$$

(3) $$P=\frac{E}{t_{d}}$$

Electrochemical impedance spectra (EIS) were obtained over the frequencies of 100 kHz and 0.1 Hz using a 0.01 V amplitude on a PEC-L01, Peccel, Yokohama, Japan.

## 3. Results

### 3.1. Characterization of Synthesized and Thermally Treated Mn-MOFs

**Figure 1:**
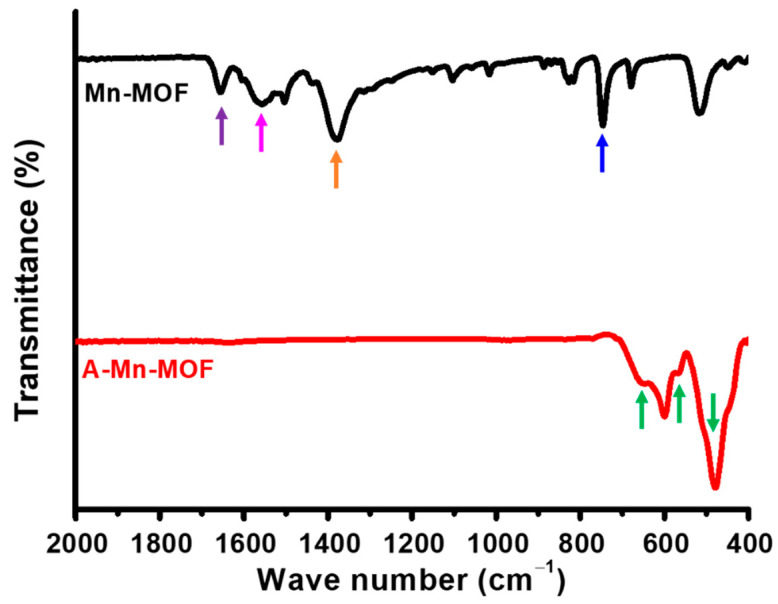
FTIR spectra of Mn-MOFs and A-Mn-MOFs.

The chemical structures of synthesized and thermally treated Mn-MOFs were observed using FTIR spectroscopy, as shown in Figure 1. For synthesized Mn-MOFs, a peak representing carbonyl C=O stretching appears at 1723 cm^−1^ (indicated by a purple arrow). It also shows distinctive bands at 1559 cm^−1^ (indicated by a pink arrow) and 1382 cm^−1^ (indicated by an orange arrow) assigned to the stretching vibration of the C-H and C-C bonds of the phenyl ring, respectively [36,37]. In addition, a peak at 747 cm^−1^ (indicated by a blue arrow) for out-of-plane C-H bending in the phenyl ring is found, confirming that the organic linker of Mn-MOFs, terephthalate, was successfully formed by the reaction between manganese chloride and terephthalic acid.

After the thermal treatment under air, it was observed that those peaks disappeared, and peaks at 652, 566, and 481 cm^−1^ are shown (indicated by green arrows), which are assigned to the characteristic Mn–O stretching vibration of Mn(III) oxide [38,39,40]. This indicates that organic linkers are decomposed, and manganese oxides are formed during the thermal treatment of Mn-MOFs in the presence of oxygen.

**Figure 2:**
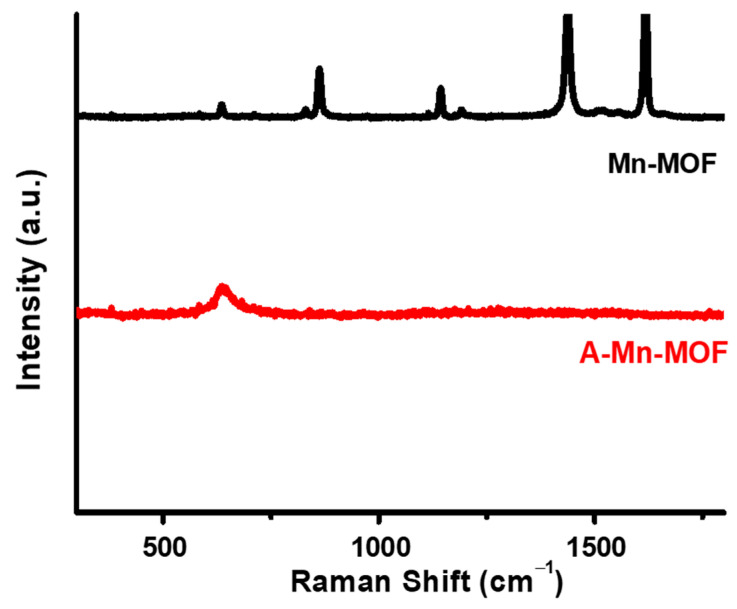
Raman spectra of Mn-MOFs and A-Mn-MOFs.

The structures of synthesized Mn-MOFs and A-Mn-MOFs were further investigated by Raman analysis, as presented in Figure 2. The Raman spectra of Mn-MOFs show strong peaks at 1435 and 1619 cm^−1^, which are assigned to CH_2_ bending and the symmetric stretching of the para-substituted benzene rings, respectively. And the peaks at 860 and 1140 cm^−1^ correspond to C-C and C-H stretching. However, these peaks are not shown for A-Mn-MOFs, resulting in the organic linkages being burned out during the thermal treatment, which is in good agreement with the FTIR results in Figure 1. Both Mn-MOFs and A-Mn-MOFs show a peak around 635 cm^−1^, which is assigned to the stretching vibration of Mn-O. The successful synthesis of Mn-MOFs and conversion into manganese oxides are confirmed by FTIR and Raman spectroscopy.

**Figure 3:**
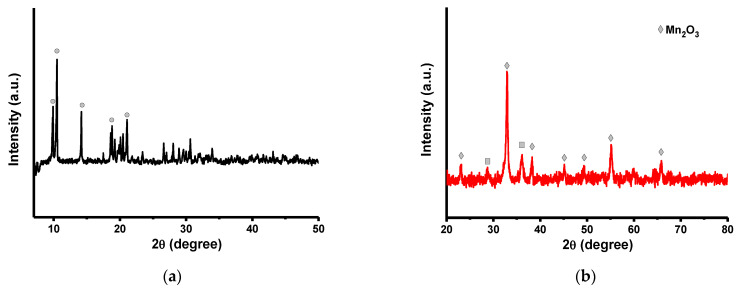
XRD patterns of (**a**) Mn-MOFs and (**b**) A-Mn-MOFs.

The crystalline nature of synthesized and thermally treated Mn-MOFs was studied by XRD spectroscopy, as shown in Figure 3. Both Mn-MOFs and A-Mn-MOFs show sharp diffraction peaks due to their high crystallinity. In Figure 3a, the diffraction peaks located at 2θ = 9.9°, 10.5°, 14.2°, 18.8°, and 18.8° (indicated by circle symbols) correspond to the (200), (010), (110), (200), and (111) planes, respectively, which is in agreement with previously reported MOFs containing terephthalate linkers [41]. The XRD pattern suggests that crystalline Mn-MOFs were successfully produced by solvothermal synthesis using manganese chloride and terephthalic acid. In Figure 3b, diffraction peaks appear in A-Mn-MOFs at 2θ = 23.0°, 32.9°, 38.2°, 45.1°, 49.3°, 55.1°, and 65.8° (indicated by diamond symbols), corresponding to the (211), (222), (400), (332), (431), (440), and (622) crystal planes of Mn_2_O_3_ with the cubic crystal phase. It was reported that Mn_2_O_3_ was synthesized by the calcination of manganese precursors, such as Mn(NO_3_)_2_ [42,43] and MnCO_3_ [44], and XRD studies showed that a cubic phase was formed. Peaks at 28.8° and 36.1° are also seen, which can correspond to the (112) and (211) planes of Mn_3_O_4_ (indicated by square symbols).

Manganese oxides show different oxidation and reduction reactions due to their diverse manganese cation oxidation states and morphology [45,46]. They generally consist of tunnel structures, resulting in the facile access and adsorption of small molecules such as electrolyte ions to the active sites. Additionally, oxygen vacancies of manganese oxide can provide additional active sites for redox reactions. These features make manganese oxides, such as Mn_2_O_3_, attractive candidates to be used as electrode materials for lithium-ion batteries or pseudocapacitors.

**Figure 4:**
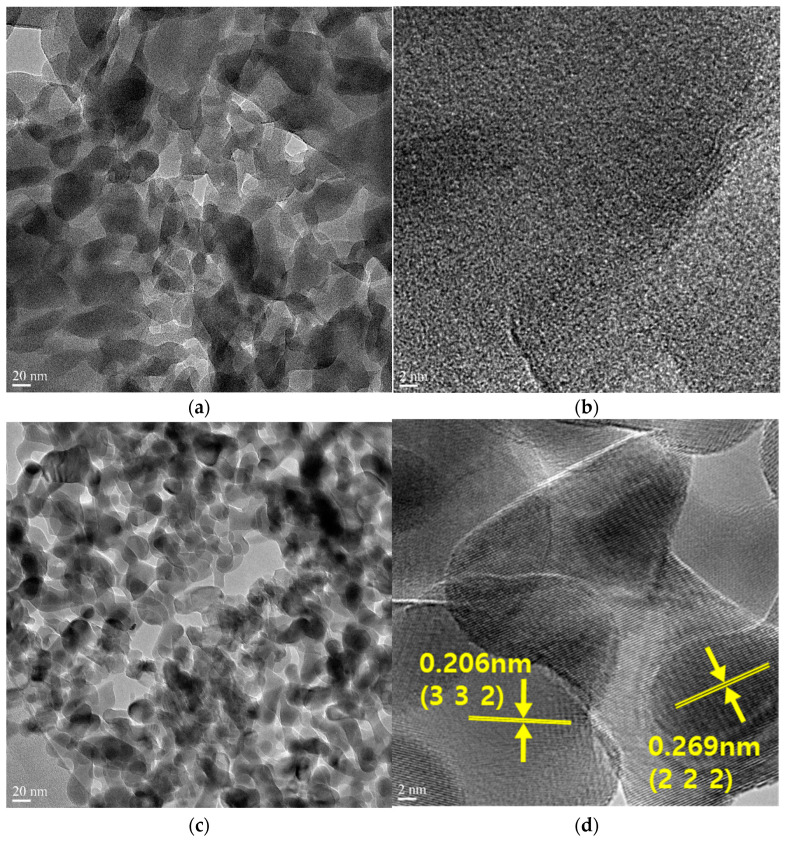
TEM images of (**a**,**b**) Mn-MOFs and (**c**,**d**) A-Mn-MOFs.

The structure features of synthesized and thermally treated Mn-MOFs were characterized by TEM, and their elemental compositions were analyzed by EDS, as shown in Figure 4 and Table 1, respectively. It is seen that the particles of the A-Mn-MOFs in Figure 4c are smaller than those of the Mn-MOFs in Figure 4a due to the decomposition caused by the thermal treatment under air. In addition, no crystalline structures are found in the Mn-MOFs. For the A-Mn-MOFs, a distinct contrast in the TEM image in Figure 4c supports the porous feature. It is also found that the interplanar distances are 0.206 and 0.269 nm, which agree with the (332) and (222) planes of cubic Mn_2_O_3_, respectively.

An EDS analysis was carried out to analyze the chemical constituents of synthesized and thermally treated Mn-MOFs. It is demonstrated that the contents of Mn, C, and O in the Mn-MOFs are 36.1, 44.7, and 19.3 wt%, respectively. For A-Mn-MOFs, the content of C was significantly decreased, since the organic linkers were burned out via thermal treatment under air. It is also shown that the Mn content relatively increases due to the removal of C.

**Figure 5:**
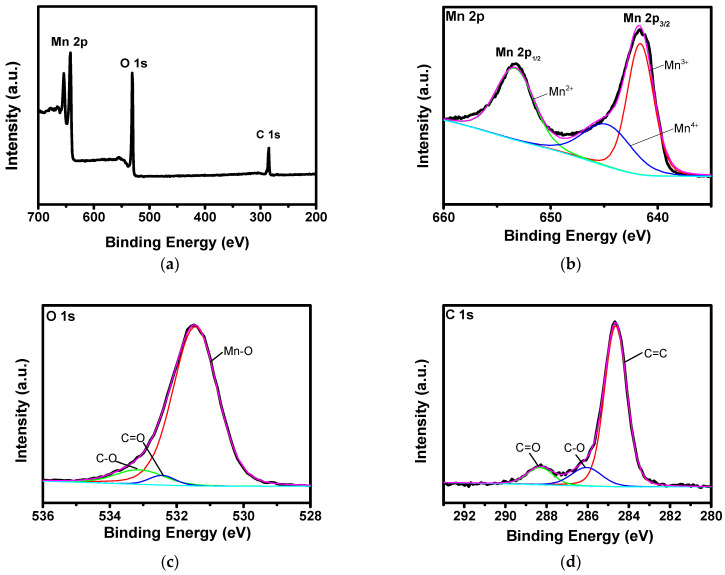
XPS spectra of A-Mn-MOFs: (**a**) survey spectrum, (**b**) Mn 2p, (**c**) O 1s, and (**d**) C 1s.

An XPS study was conducted to investigate the chemical oxidation states and compositions of A-Mn-MOFs, as shown in Figure 5. A survey scan in Figure 5a shows the peaks for Mn 2p, O 1s, and C 1s without any impurity. Figure 5b displays the binding energy spectrum of Mn 2p, and it is seen that the spin-orbital energy separation between the characteristic peaks for Mn 2p_3/2_ and Mn 2p_1/2_ is 11.7 eV, which demonstrates that the manganese in A-Mn-MOFs mainly exists in the Mn(III) oxidation state. The peaks at 641.8 eV and 645.2 eV are assigned to Mn(III) and Mn(II), respectively. The presence of Mn(III) confirms the formation of Mn_2_O_3_, which was also found in the FTIR spectrum. The O 1s spectra in Figure 5c shows a main peak of Mn-O with two slight peaks of C-O and C=O, and the C 1s spectra are indicative of carbon corresponding to C=O, C-O, and C-C, as shown in Figure 5d, which can be assigned to the carbonate, terephthalate, produced via the thermal treatment of organic linkers.

**Figure 6:**
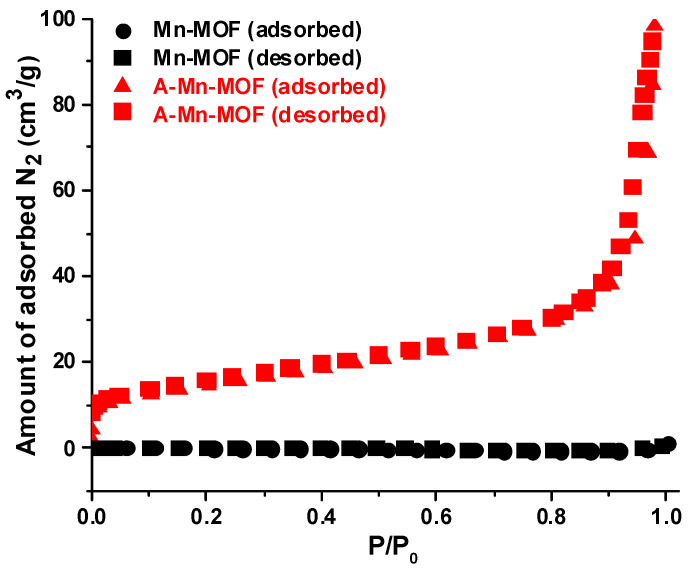
N_2_ adsorption (closed symbols)/desorption (open symbols) isotherms.

The surface area and porosity of the synthesized and thermally treated Mn-MOFs were investigated by measuring the nitrogen adsorption/desorption isotherms, as shown in Figure 6, and Table 2 shows the pore characteristics. It was noted that thermal treatment improves the specific surface area and pore volumes of Mn-MOFs. The A-Mn-MOFs have a surface area and total pore volume of 50.92 m^2^ g^−1^ and 0.15 cm^3^ g^−1^, respectively, which are 100 times higher than those of pristine Mn-MOFs. As seen in the EDS analysis, organic linkers were burned out via thermal treatment in the presence of oxygen, resulting in the release of gaseous molecules, which can create pores in A-Mn-MOFs. It is also noticeable that most of the pores in A-Mn-MOFs are mesopores, and this feature is beneficial for surface-induced pseudocapacitance owing to the large interfacial area between electrodes and electrolytes. It can be expected that applying highly porous A-Mn-MOFs to supercapacitor electrodes facilitates the access of electrolyte ions, promoting energy storage performance. Also, it is proven that thermally treating MOFs under air is a successful way to produce porous metal oxides for supercapacitor electrodes.

Surface properties are some of the most critical factors to determine the energy storage performance of supercapacitor electrodes since chare storage occurs through electrostatic adsorption at the interface between the electrodes and electrolytes. Ragupathy et al. reported the synthesis of nanostructured MnO_2_ with a high surface area by using exothermic redox reaction [47]. Chen et al. reported the synthesis of porous MnO_2_ nanorods using mesoporous silica as a template [48]. Another strategy is utilizing porous carbon to overcome the limited surface porosity and electrical conductivity of manganese oxides [49,50,51,52].

### 3.2. Electrochemical Properties of Synthesized and Thermally Treated Mn-MOFs

The electrochemical properties of synthesized and thermally treated Mn-MOFs were measured in a three-electrode system with Pt as a counter electrode, Ag/AgCl as a reference electrode, and 1 M sodium sulfate aqueous solution as an electrode. The working electrode was prepared by drop-casting synthesized and thermally treated MOFs, PVDF, and carbon black dispersed in NMP on the graphite foil. CV and GCD curves were obtained to study energy storage performance.

**Figure 7:**
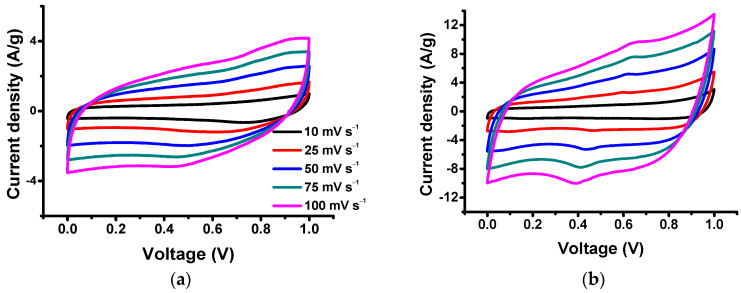
CVs of (**a**) Mn-MOF and (**b**) A-Mn-MOF electrodes.

Figure 7 shows the CV curves of the MOF electrodes at scan rates varying from 10 to 100 mV s^−1^. Both electrodes exhibit rectangular CVs, which are typical for supercapacitors, with shallow redox reaction humps. Also, it is demonstrated in Figure 7b that the thermally treated Mn-MOFs have a higher integrated area of CVs over the whole voltage range, suggesting higher electrochemical capacitances.

**Figure 8:**
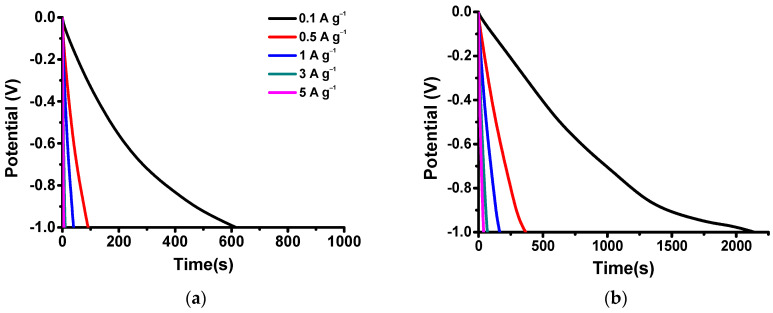
Discharge curves of (**a**) Mn-MOF and (**b**) A-Mn-MOF electrodes.

Galvanostatic charge–discharge testing was performed in the current density range from 0.1 to 5 A g^−1^, and the energy storage performance, including the specific capacitance and energy and power densities, were calculated from the discharge curves from 0 to −1 V, as shown in Figure 8 and Table 3. The A-Mn-MOFs exhibit a significantly longer discharge time, which results in significantly higher specific capacitances and energy densities. The specific capacitances of Mn-MOFs and A-Mn-MOFs are 61.6 and 214.0 F g^−1^ at 0.1 A g^−1^, respectively. It is also seen that the energy densities of the Mn-MOFs and A-Mn-MOFs are 8.6 and 29.7 Wh kg^−1^, respectively. The power density of A-Mn-MOFs (2.35 kW kg^−1^ at 5 A g^−1^) is also higher compared to that of Mn-MOFs (1.91 kW kg^−1^ at 5 A g^−1^) due to a lower IR drop.

Pseudocapacitors store their energy via redox reactions through faradaic electron transfer to metal centers via the intercalation or adsorption of ions similar to batteries, while their reaction kinetics are fast, which is a characteristic of EDLCs. Peak-shaped CVs are obtained for A-Mn-MOFs, as observed in Figure 7b, due to electronic transitions between localized energy levels like battery electrodes [53]. The discharge curves for A-Mn-MOFs have some humps (steady potential–time plots) due to the phase changes of the manganese oxides via faradaic redox reactions, which are also characteristics of batteries. It is known that pseudocapacitive behavior originates from intercalation, underpotential deposition, and rapid surface redox reactions, and Figure 7b shows typical galvanostatic discharging signatures for pseudocapacitors with intercalation, including partially reversible redox reactions [54]. The redox peaks in CVs with non-linear discharge curves clearly indicate that A-Mn-MOFs have pseudocapacitive behaviors, resulting in superior energy storage performance.

**Figure 9:**
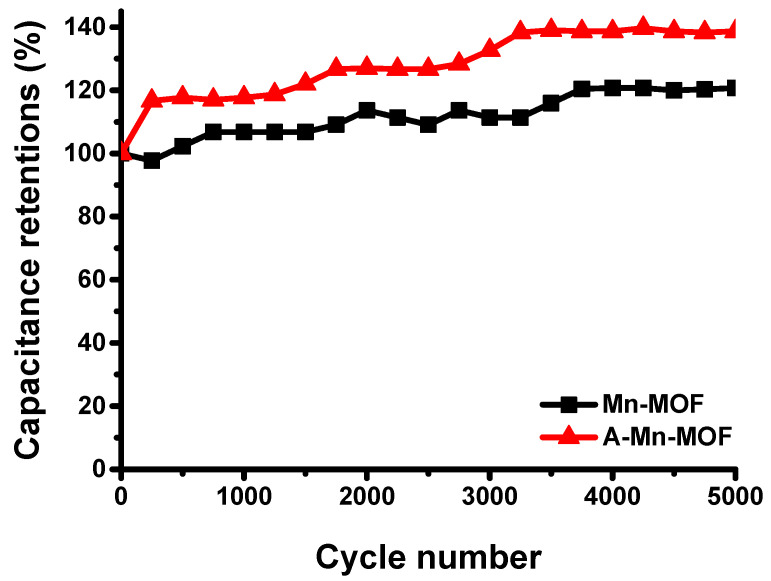
Cyclic performance of Mn-MOF and A-Mn-MOF electrodes.

To investigate cyclic stability, the capacitance retention was measured by GCD testing at a current density of 1 A g^−1^. Figure 9 shows that the capacitance retention for Mn-MOF and A-Mn-MOF electrodes constantly increased during 3000 cycles, reaching 120% for Mn-MOFs and 139% for A-Mn-MOFs. Then, these values were maintained until 5000 cycles were reached.

**Figure 10:**
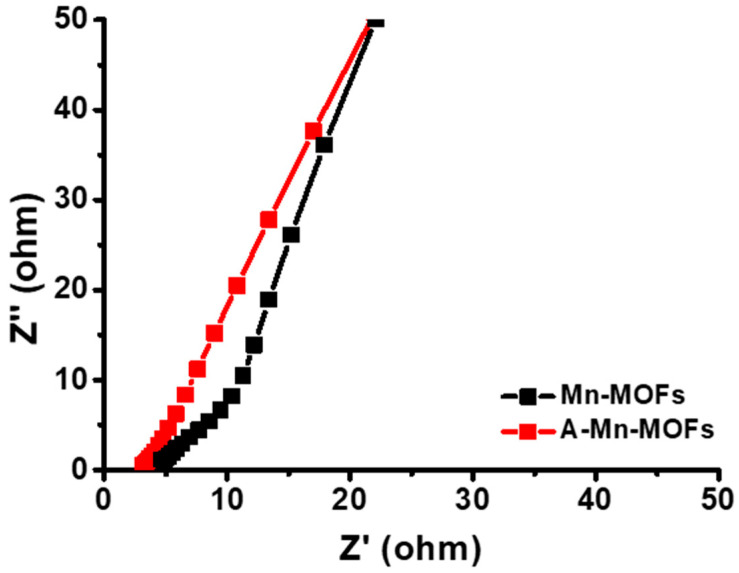
Nyquist plots of Mn-MOF and A-Mn-MOF electrodes.

The EIS was measured to further evaluate the electrochemical performance, as presented in Figure 10. The Nyquist plots exhibit a straight line, suggesting a low charge transfer resistance, which is a characteristic of pseudocapacitor electrodes [55]. It is seen that the electrolyte resistance (R_s_) values of Mn-MOFs and A-Mn-MOFs is 4.6 Ω and 2.9 Ω, respectively. In addition, the slope of A-Mn-MOFs is higher than that of Mn-MOFs, which represents facile ion diffusion across the diffuse layer due to the larger surface porosity.

Most studies on supercapacitor electrodes focus on porous carbon materials, such as graphene, carbon nanotubes (CNTs), or carbon nanofibers (CNFs) [56,57,58,59,60]. However, these are based on electrochemical double-layer capacitances with electrostatic charge storage at the interface between electrodes and electrolytes, showing limited specific capacitances and energy densities. Transition metal oxides are good candidates to overcome this limitation by inducing faradaic redox reactions, known as pseudocapacitance.

It has been reported that thermally treated MOFs can produce porous metal oxide electrodes for storage devices [61,62]. Porous Mn_2_O_3_ was prepared by the synthesis and heat treatment of Mn-MOFs for anodes in lithium-ion batteries, which provided higher energy densities due to their high porosity and low potential of Mn. Bai et al. reported the synthesis of porous Mn_2_O_3_ through the calcination of [Mn(tetrabromoterephthalate)(4,4′-bipyridine)(H_2_O)_2_]_n_ at 600 °C, and they exhibit superior electrochemical performance including a high specific capacity, long cycling stability, and good rate property [63]. Also, Zheng et al. prepared hollow Mn_2_O_3_ microspheres through the pyrolysis of Mn-MOFs at 450 °C, which were synthesized using trimesic acid as an organic linker [64]. They exhibited improved capacity and excellent cycling stability due to the reduced diffusion length of lithium ions and electrons.

Table 4 shows the comparison of the energy storage performance of manganese oxide-based electrodes, and it can be seen that the capacitance value of A-Mn-MOF is higher than that of previously reported materials. The high energy storage performance of A-Mn-MOFs in this work may be attributed to their high surface porosity, which promotes the access of electrolyte ions to the electrode surface.

## 4. Conclusions

Tuning the electrochemical and surface properties of electrode materials is considered the most effective way to improve the energy storage performance of supercapacitors. Porous manganese oxides were chosen as supercapacitor electrodes due to their redox-active features with electrolyte accessibility. They were synthesized through the thermal treatment of synthesized Mn-MOFs consisting of manganese ions linked by organic bridging ligands at 400 °C under air. The successful synthesis of Mn-MOFs was confirmed by FTIP and Raman spectroscopy, and thermal conversion to Mn_2_O_3_ was confirmed by XRD spectroscopy, XPS, and TEM. It was also seen that the specific surface area and total pore volume of the A-Mn-MOFs were 50.92 m^2^ g^−1^ and 0.15 cm^3^ g^−1^, respectively, which were 100 times higher than those of pristine Mn-MOFs, confirming that highly porous metal oxides can be synthesized by thermally treating MOFs. The energy storage performance was investigated, and redox reaction humps were found in the CVs and discharge curves of A-Mn-MOFS, which are indicative of pseudocapacitive behavior. As a result, the A-Mn-MOFs showed a high specific capacitance of 214.0 F g^−1^ and energy and power densities of 29.7 Wh kg^−1^ and 2.51 kW kg^−1^ at 0.1 A g^−1^, respectively, with a capacitance retention of 139%. Their superior energy storage performance with a large surface porosity concluded that porous Mn_2_O_3_ synthesized by thermally treating MOFs under air is a promising electrode material for pseudocapacitors.

## Figures and Tables

**Table 1:** Compositions of Mn-MOFs and A-Mn-MOFs analyzed by TEM/EDS.

Element	Mn-MOFs	A-Mn-MOFs
Mn	36.1 wt%	72.7 wt%
C	44.6 wt%	4.1 wt%
O	19.3 wt%	23.2 wt%

**Table 2:** Structural parameters of Mn-MOFs and A-Mn-MOFs.

	SSA^1^ (m^2^ g^−1^)	TPV ^2^ (cm^3^ g^−1^)	V_micro_ ^3^ (cm^3^ g^−1^)	V_meso_ ^4^ (cm^3^ g^−1^)
Mn-MOFs	0.49	1.04 × 10^−3^	9.92 × 10^−5^	9.41 × 10^−4^
A-Mn-MOFs	50.92	0.15	2.84 × 10^−3^	0.15

^1^ SSA: specific surface area. ^2^ TPV: total pore volume. ^3^ V_micro_: micro-pore (<2 nm) volume. ^4^ V_meso_: meso-pore (2–50 nm) volume.

**Table 3:** Electrochemical properties of Mn-MOF-based electrodes.

	Mn-MOFs	A-Mn-MOFs
*C*_sp_ (F g^−1^) at 0.1 A g^−1^	61.6	214.0
*E* (Wh kg^−1^) at 0.1 A g^−1^	8.6	29.7
*P* (kW kg^−1^) at 5 A g^−1^	1.91	2.35

**Table 4:** Specific capacitances of manganese oxide-based electrodes reported earlier.

Materials	Electrolyte	Current Density	Specific Capacitance	Reference
MnO_2_ nanorods	1 M Na_2_SO_4_	0.5 A g^−1^	163.5 F g^−1^	[31]
MnO_2_ nanoparticles	1 M KOH	0.5 A g^−1^	175.5 F g^−1^	[65]
Mn_3_O_4_ thin films	2 M KOH	0.5 mA cm^−2^	109 F g^−1^	[66]
Mn_3_O_4_ nanoparticles	1 M KCL	0.5 A g^−1^	144.5 F g^−1^	[67]
Mn_2_O_3_ nanocubes	0.5 M Na_2_SO_4_	0.1 A g^−1^	191.1 F g^−1^	[32]
Mn_2_O_3_ nanorods	1 M NaCl	0.5 A g^−1^	93 F g^−1^	[68]
A-Mn-MOFs	1 M Na_2_SO_4_	0.1 A g^−1^	214.0	This Work
		0.5 A g^−1^	181.2	

## Data Availability

Data are contained within the article.
